# Procedural Learning and Retention in Older Subjects: The Contribution of Sensorimotor and Premotor Cortex

**DOI:** 10.1002/brb3.71845

**Published:** 2026-10-16

**Authors:** Christopher E. J. Doppler, Linda Meyer, Ralph Weidner, Peter H. Weiss, Gereon R. Fink

**Affiliations:** ^1^ Cognitive Neuroscience, Institute of Neuroscience and Medicine (INM‐3) Forschungszentrum Jülich Jülich Germany; ^2^ Department of Neurology, Faculty of Medicine University Hospital Cologne, University of Cologne Cologne Germany; ^3^ Department of Psychiatry Alexius/Josef Hospital Neuss Germany

**Keywords:** motor learning, motor skill acquisition, motor skill learning, serial reaction time task

## Abstract

**Purpose:**

To investigate the neurobiological basis of procedural learning and retention of motor memories in aging.

**Method:**

We used a modified serial reaction time (SRT) task with feedback in two age groups: young (*n* = 18, 24.9 ± 3.9 years, range: 18–32 years) and older (*n* = 19, 63.5 ± 5.1 years, range: 54–73 years) healthy participants. We assessed procedural learning while participants underwent functional magnetic resonance imaging (fMRI, Day 1). On the following day, we assessed retention behaviorally outside the scanner.

**Finding:**

Both groups showed significant procedural learning (*p* < 0.001) and retention (*p* < 0.05). Notably, retention was enhanced in older compared to younger adults (*p* < 0.01), even after normalizing the reaction time (RT) gains to individual RT (*p* < 0.05). Functional imaging revealed more robust recruitment of the left sensorimotor cortex (SM1) during procedural learning in older participants. Besides, left premotor cortex activity during procedural learning predicted retention in both age groups.

**Conclusion:**

Our data show that procedural learning was preserved in healthy older adults. At the same time, retention was even enhanced in healthy older adults. These processes were associated with activation changes in the contralateral sensorimotor and premotor cortices.

## Graphical abstract

Procedural learning is preserved, and consolidation is enhanced in healthy older adults. These behavioral effects are associated with increased recruitment of contralateral sensorimotor and premotor cortices during learning.

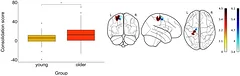

## 1 Introduction

Procedural learning, consolidation, and retention are crucial for human development, constituting the foundation of learning motor skills like playing an instrument or riding a bike. In aging, these processes become particularly important for the rehabilitation of motor deficits, especially in age‐related conditions such as stroke.

The serial reaction time (SRT) task has been widely used to examine (procedural) learning, consolidation, and retention of motor (sequence) knowledge (Nissen and Bullemer 1987). In the SRT task, participants respond to visual cues by pressing a corresponding button. Unbeknownst to the participants, some trials follow a predetermined underlying sequence, whereas in others a random succession of cues is presented. The improvement in reaction time (RT) from random to sequence‐based successions is considered a parameter of procedural learning.

For procedural learning, different phases have been described: an initial fast phase with rapid improvements within sessions and a slow phase with further improvements across different sessions (Dayan and Cohen 2011; Doyon et al. 2003). Consolidation results in a stabilization of motor memories. During the fast phase, a distributed network of brain areas is activated, with the primary sensorimotor cortex (SM1) playing a central role (Dayan and Cohen 2011; Hardwick et al. 2013). Moreover, the premotor cortex is particularly relevant for SRT performance and consolidation of motor memories in general (Hardwick et al. 2013; Kornysheva and Diedrichsen 2014; Robertson 2007).

There is abundant research on how procedural learning, consolidation, and retention change during healthy aging, with many studies using the SRT task (for a review, see Zwart et al. 2019). However, thus far, the published studies have yielded conflicting results. For procedural learning (in particular for sequence‐specific learning [SSL] in the SRT task), many studies showed similar performance levels in young and older participants (Bhakuni and Mutha 2015; Daselaar et al. 2003; Fraser et al. 2009; Frensch and Miner 1994; Gaillard et al. 2009; Howard and Howard 1989, 1992; Meissner et al. 2016; Nemeth and Janacsek 2011). In contrast, other studies reported an age‐related decline in procedural learning attributed to higher task complexity, for example, when using higher order sequences (Howard et al. 2004). Notably, in most studies, consolidation was worse in older adults compared with healthy young adults (Brown et al. 2009; Nemeth and Janacsek 2011; Spencer et al. 2007; Wilson et al. 2012). Although several studies have examined the neurobiological basis of procedural learning (Daselaar et al. 2003; Durand‐Ruel et al. 2023; King et al. 2018), consolidation (Fitzroy et al. 2021; Fogel et al. 2014; King et al. 2017), and retention (Berghuis et al. 2019) in aging, only a few of them have incorporated feedback during the learning phase (Durand‐Ruel et al. 2023; King et al. 2018).

Therefore, this study aimed to extend current knowledge of procedural learning and retention by examining the effects of aging and their neurobiological underpinnings using a modified version of the SRT task combined with functional MRI. Unlike previous studies, we provided direct trial‐by‐trial feedback to participants, hypothesizing that this would facilitate performance. Participants performed the task on two subsequent days. On Day 1, we studied procedural learning and its neural mechanisms using fMRI, followed by a behavioral retention assessment on Day 2. On the basis of the studies mentioned above, we hypothesized that young and older participants would exhibit similar performance in procedural learning. However, we expected older participants to show worse retention. Furthermore, we hypothesized that these effects would be associated with changes in activity in essential motor areas.

## 2 Methods

### 2.1 Participants

We recruited 21 young and 27 older participants. Sample size was determined based on a previous study with a similar design (Dovern et al. 2011). All participants had normal or corrected‐to‐normal vision, and none had a history of neurological or psychiatric disease or were taking psychopharmacologically active medication. Handedness was determined using Oldfield's laterality quotient (Oldfield 1971), and left‐handers were excluded. Both groups were tested for clinically relevant symptoms of depression using the Beck Depression Inventory (BDI) (Beck et al. 1996), executive functions were tested using the Trail Making Test A and B (Arnett and Labovitz 1995), and visuospatial working memory was examined using the Corsi block‐tapping task (Corsi 1973). Impulsivity was assessed using the Barratt Impulsiveness Scale (BIS‐11) (Preuss et al. 2008). The Cambridge Behavior Scale was used to determine participants’ Empathy Quotients (Baron‐Cohen and Wheelwright 2004). Additionally, the older group underwent a dementia screening using the Mini‐Mental State Examination (MMSE) (Folstein et al. 1975). All older participants scored above the cutoff in the MMSE (≥27 of 30 points). One older participant, in whom the BDI indicated clinically relevant signs of depression, was excluded from the study. For the included participants, BDI scores did not differ significantly between age groups (young: 4.7 ± 3.5, older: 3.4 ± 3.0; *W* = 102, *p* = 0.33).

Eleven participants had to be excluded due to extensive head movements (>3 mm) during the functional MRI measurement or due to other MRI artifacts (*n* = 3), an error rate >2 standard deviations (SD) above the mean (*n* = 4), structural brain abnormalities (i.e., silent infarction, atrophy; *n* = 2), clinically relevant signs for depression (*n* = 1), or lack of understanding of the task (*n* = 1). Thus, data from 18 young (6 women; age [mean ± SD]: 24.9 ± 3.9, range: 18–32 years) and 19 older participants (8 women; age [mean ± SD]: 63.5 ± 5.1, range: 54–73 years) entered final analyses. Behavioral data of the second day of the experiment from one participant of the young group are missing due to his decision to withdraw from the study.

### 2.2 Stimuli and Experimental Paradigm

For the current fMRI study, we used a modified SRT task (Figure 1). Participants saw three grey boxes arranged horizontally. A cross appeared in one of these boxes on each trial. Using a Lumitouch keyboard, they were instructed to press the button that spatially corresponded to the location of the cross as fast as possible with the fingers of their (dominant) right hand. Unbeknownst to the participants, stimuli were presented either following predetermined sequences (S) or in a pseudo‐randomized order (R).

**FIGURE 1:**
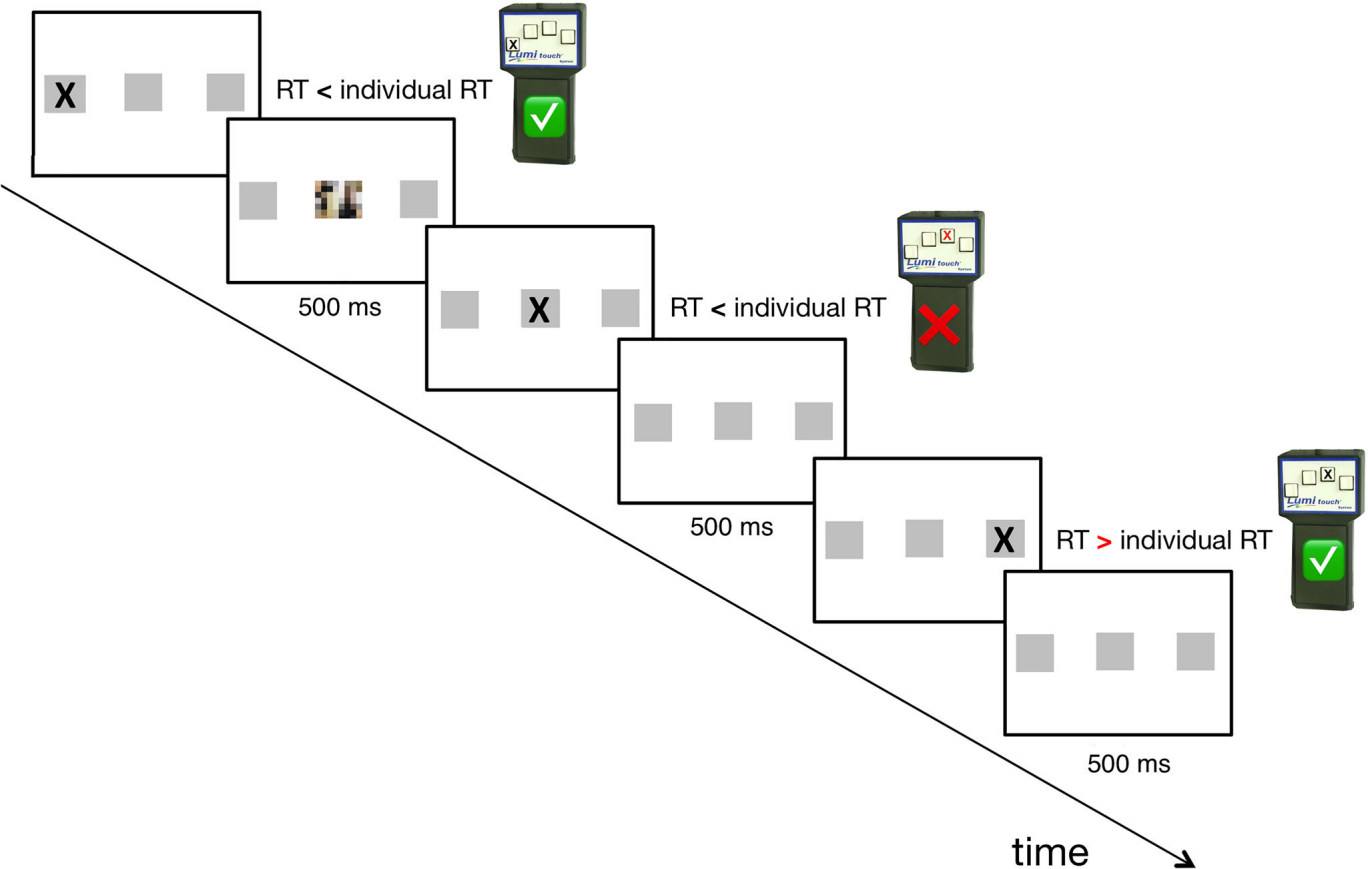
**Timeline of the modified version of the serial reaction time (SRT) task on Day 1**. During a practice session before the experiment, each participant's individual reaction time (RT) threshold was determined. If the participant pressed the spatially corresponding button faster than this individual RT threshold, the respective feedback was displayed for 500 ms, followed by a 500 ms inter‐stimulus interval. If the participant responded with a spatially non‐corresponding button or responded more slowly than their individual RT threshold, no feedback was provided.

Prior to the fMRI measurement, each participant's median RT was assessed during a practice session outside the MRI scanner. During the fMRI task, feedback was displayed on a screen whenever participants pressed the correct button faster than their individually determined median RT. If the participants pressed the wrong button or the correct button slower than their median RT, no feedback was provided.

We used three different nine‐element sequences for each participant. All sequences for the modified SRT task—also the ones in the pseudo‐randomized blocks—were constructed using the following rules to avoid possible confounds due to different structures of the sequences (as in Dovern et al. 2011):

1. Two repeats of one stimulus location at most (e.g., not … 111 …).
2. Two unidirectional stimulus locations in a row at most (e.g., not 123 …).
3. No repeated doublets (e.g., not 2323 …).
4. No three consecutive repetitions (e.g., not 331,122 …).
5. Each stimulus location occurred three times in every sequence, each time followed by a different stimulus location.

The most significant modification of the current SRT task compared to standard versions was that we provided direct (i.e., on each trial) feedback to our participants. The duration of the feedback, as well as the inter‐stimulus interval, was 500 ms. To minimize potential biases due to a given feedback type, for example, monetary reward or punishment (Wächter et al. 2009), we used three different types of feedback stimuli in every participant (a €50 banknote, a photo of the investigator giving positive feedback (“thumbs up”), and a scrambled image of a combination of the other two stimuli) and collapsed the data across feedback types. Each run contained 16 experimental blocks: R‐R‐S‐S‐S‐S‐S‐S‐S‐S‐S‐S‐R‐R‐S‐S (see Figure 2A). The feedback was presented during the first ten blocks of each session. The order of feedback types and their combination with the nine‐element sequences were pseudo‐randomized. As a visual baseline, the stimulus was shown alternating with a blank screen for one block (i.e., 30 s) before starting the task for the respective feedback type. During fMRI data acquisition, RTs and manual responses were recorded. We used a block length of around 30 s to maximize the efficiency of the blocked fMRI design. The block length resulted in three repetitions of each of the 9‐element sequences per fMRI block, for a total of 36 repetitions of each of the sequences in total (12 fMRI blocks with a sequential order of stimuli × 3 sequence repetitions per fMRI block).

**FIGURE 2:**
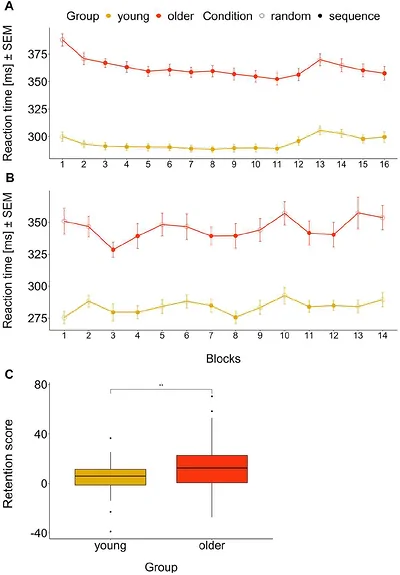
**Main behavioral results**. (A) Median reaction times (RT) on Day 1 per experimental block for the group of young (*n* = 18, yellow lines) and older (*n* = 19, orange lines) participants. (B) Median RT on Day 2 per experimental block for both groups. Empty circles represent blocks with (pseudo‐)random order of cues, and filled circles represent blocks with an underlying sequential order. (C) Retention score for young and older participants (yellow and red boxes, respectively). Retention scores are operationalized by subtracting the mean of the median RTs of the sequence blocks for each motor sequence from the mean of the median RTs of all random blocks on Day 2 of the experiment. Asterisks denote significant differences between groups (**p < 0.01).

On Day 2, a shortened version of the same task (R‐R‐S‐S‐R‐R‐S‐S‐R‐R‐S‐S‐R‐R) was performed (Figure 2B). Each of the three motor sequences was presented in two consecutive sequence blocks, resulting in six sequence blocks in total. The two blocks of a given sequence were separated from the following two blocks by two random blocks. We used the median RTs of sequence and random blocks to compute the retention score (see below). No feedback was given during this second phase of retrieval, assuring that the mere recall of the sequences, representing the effect of retention, was examined.

### 2.3 FMRI Data Acquisition and Preprocessing

We used a 3.0 T Trio MR‐scanner (Siemens, Erlangen, Germany) to obtain T2*‐weighted gradient echo‐planar images (EPI) with BOLD contrast (matrix size: 64 × 64; voxel size: 3.1 × 3.1 × 3.5 mm^3^; field of view: 200 mm; repetition time: 2200 ms; echo time: 30 ms; flip angle: 90°). Using interleaved slice acquisition, each volume contained 36 axial slices. Anatomical T1‐weighted 3D MP‐RAGE images with a voxel size of 1 × 1 × 1 mm^3^ were acquired for anatomical reference. The first six EPI volumes were discarded to control for T1 equilibration effects. FMRI data were analyzed using the Statistical Parametric Mapping software SPM12 (Wellcome Department of Imaging Neuroscience, London; http://www.fil.ion.ucl.ac.uk/spm). Images were spatially realigned, and the segmentation function was used for spatial normalization after computing a mean EPI image for each subject. The resulting images were smoothed using an 8 mm full‐width half‐maximum (FWHM) Gaussian kernel.

Image beta values were extracted using REX (version alpha 0.5, https://www.nitrc.org/projects/rex) and MarsBaR (version 0.44, http://marsbar.sourceforge.net).

### 2.4 Statistical Analysis

#### 2.4.1 Statistical Analysis of the Behavioral Data

The behavioral data were analyzed using RStudio (Version 2024.12.0) and the package lme4 (Bates et al. 2015). The first trial of each block, error trials, and the trials after error trials were removed to account for post‐error slowing. To minimize the effect of potential RT outliers, we calculated the median RT per block and per participant. Error rates were measured as the percentage of error trials relative to all trials in the respective block.

Procedural learning was assessed by analyzing error rates and RT acquired during scanning on Day 1 using linear mixed models. Models were developed using a stepwise approach, beginning with an intercept‐only model and progressively adding predictors. The final models contained the factors “condition” (sequence or random blocks) and “group” (young or older adults) as fixed effects and “subject” as the random effect. As in previous studies (Dovern et al. 2011), performance (in our case, retention) was operationalized by subtracting the mean of the median RT of the sequence blocks for each motor sequence from the mean of the median RT of all random blocks on Day 2 of the experiment. We deliberately chose this operationalization of retention rather than a more traditional approach to minimize potential bias in RT when comparing different paradigm settings within (Day 1) and outside (Day 2) the scanner.

Unless stated otherwise, further group comparisons were performed using Welch's *t*‐test. Significance was accepted at *p* < 0.05. All descriptive statistics are provided as mean ± SD.

#### 2.4.2 Statistical Analysis of the fMRI Data

For the first‐level analysis in SPM12, the onset and duration of the experimental conditions were used as regressors. Head movement parameters were added as regressors of no interest. The visual baseline was modeled implicitly. We calculated the first‐level baseline contrasts by comparing each regressor to the implicit baseline, setting the regressor of interest to 1 and all others to 0. The BOLD response was modeled using a canonical hemodynamic response function and its first derivative.

At the second level, these contrasts were analyzed using a mixed‐design analysis of variance (ANOVA, full factorial design in SPM12) with the between‐subject factor group (young, older adults) and the within‐subject factors condition (random, sequence) and feedback (yes, no).

Finally, we performed a whole‐brain regression analysis to test the hypothesis that activity in core motor areas during procedural learning on Day 1 is associated with individual differences in retention, as assessed behaviorally on Day 2. Differential first‐level contrast images for the main effect of condition (sequence > random) for each subject entered the regression analysis, and the behavioral retention score was used as a covariate. We identified brain regions co‐varying with the individual's retention by applying a directed *t*‐contrast.

Significant activations are reported using the nested strategy recommended by Friston et al. (1996). Thus, activations meeting either a cluster‐wise or a voxel‐wise correction for multiple comparisons are considered significant. Specifically, for cluster‐wise correction, a statistical threshold of *p* < 0.05, family‐wise error (FWE) corrected for multiple comparisons, was used at the cluster level based on a cluster‐defining threshold of *p* < 0.001, uncorrected (Poline et al. 1997; Woo et al. 2014). A statistical threshold of *p* < 0.05, FWE corrected for multiple comparisons at the voxel level, was adopted for voxel‐wise correction (Flandin and Friston 2019).

For plotting the fMRI clusters, we used Nilearn (https://nilearn.github.io) and MRIcro (Version 1.9.0, https://www.mccauslandcenter.sc.edu/crnl/mricro). The SPM Anatomy toolbox (Version 3) was used to identify the corresponding brain regions (Eickhoff et al. 2005).

## 3 Results

### 3.1 Neuropsychological Data

Screening for dementia in the older group using the MMSE did not reveal any pathological results. BDI did not differ significantly between groups (*W* = 101.5, *p* = 0.33). As expected, older participants performed worse than young participants in the Corsi Block‐Tapping Task (Block Span: older group: 5.1 ± 0.8, young group: 6.4 ± 1.2, *t*(32) = 3.71, *p* < 0.001) and in both Trail Making Tests (Trail Making Test A: 37.7 ± 20.2 s, young group: 23.4 ± 7.4 s, *t*(33) = 2.69, *p* < 0.05; Trail Making Test B: 77.2 ± 20.4 s, young group: 44.0 ± 11.6, *t*(33) = 5.76, *p* < 0.001). In contrast, the quotient of Trail Making Test A and B did not differ significantly between groups (young group: 2.1 ± 0.7, older group: 2.3 ± 0.7, *t*(33) = 1.08, *p* = 0.287). Moreover, there were no group differences regarding the Empathy Quotient according to the Cambridge Behavior Scale (young group: 38.2 ± 10.9, older group: 39.8 ± 12.4, *t*(34) = 0.40, *p* = 0.693) and Impulsivity according to the BIS (young group: 56.6 ± 8.0, older group: 54.3 ± 7.9, *t*(32) = 0.40, *p* = 0.402).

### 3.2 Behavioral Data

#### 3.2.1 Procedural Learning

On Day 1 of the experiment, procedural learning was assessed. Error rates were significantly lower in sequence compared to random blocks (*β* = −0.95, standard error (SE) = 0.36, *t*(37.01) = −2.64, *p* < 0.05) and in older compared to younger participants (*β* = −2.94, SE = 0.93, *t*(37.00) = −3.17, *p* < 0.01).

RT data (Figure 2A) showed significantly shorter RT in sequence blocks compared to random blocks (*β* = −8.66, SE = 1.76, *t*(1739.00) = −4.94, *p* < 0.001), implying successful procedural learning, longer RT in older participants (*β* = 72.92, SE = 8.78, *t*(40.46) = 8.30, *p* < 0.001), and a significant interaction between factors condition and group (*β* = −5.83, SE = 2.45, *t*(1739) = −2.38, *p* < 0.05). This interaction was driven by the fact that the mean difference in RT between random and sequence blocks was greater in older (14.5 ms) than in the younger (8.7 ms) participants. Notably, when normalized to baseline performance during the first random block, RT improvements between the sequence block at the end and the beginning of the training did not differ significantly between groups (*t*(34.9) = 1.26, *p* = 0.21). Moreover, no significant group differences were observed in potential motor habituation effects, assessed as RT improvements between the random block at the end and the beginning of the training (*t*(35.0) = 1.92, *p* = 0.06).

#### 3.2.2 Retention

RT data from Day 2 of the experiment are shown in Figure 2B. Significant retention was observed for the older adults (13.8 ± 19.6 ms, *t*(18) = 4.92, *p* < 0.001) and the young adults (5.1 ± 12.6 ms, *t*(16) = 2.74, *p* < 0.05). Comparing the retention scores of the two groups, the older participants outperformed the younger participants in retaining motor sequence knowledge (Figure 2C, *t*(34) = 2.74, *p* < 0.01).

Potentially, better retention in older participants could have been biased by their slower RTs, leading to a larger nominal difference in RT between sequence and random blocks. Therefore, we also calculated the RT gain in sequence blocks relative to the average individual RT in random blocks. In older participants, this parameter, representing the retention effect normalized to RT in random blocks, was also higher than in younger participants (3.4% ± 2.9% vs. 1.4% ± 2.1%, *t*(34) = 2.34, *p* < 0.05). Moreover, the change in RT for random blocks between the 2 days of the experiment did not differ significantly between groups, suggesting that this retention effect was not influenced by mere habituation to the motor task in young participants (*β* = 5.85, SE = 3.78, *t*(696.10) = 1.55, *p* = 0.122).

### 3.3 Functional Imaging Data

#### 3.3.1 Main Effect of Group

Comparing both groups (contrast young > older adults, Figure 3A), young participants showed greater activation of the inferior parietal lobule bilaterally and the left middle temporal gyrus. For the inverse contrast (older > young adults, Figure 3B), a bilateral network comprising the precentral gyrus, the inferior and superior parietal lobules, the inferior frontal gyri, the left inferior temporal lobe, and the left middle occipital gyrus was activated (Table 1).

**FIGURE 3:**
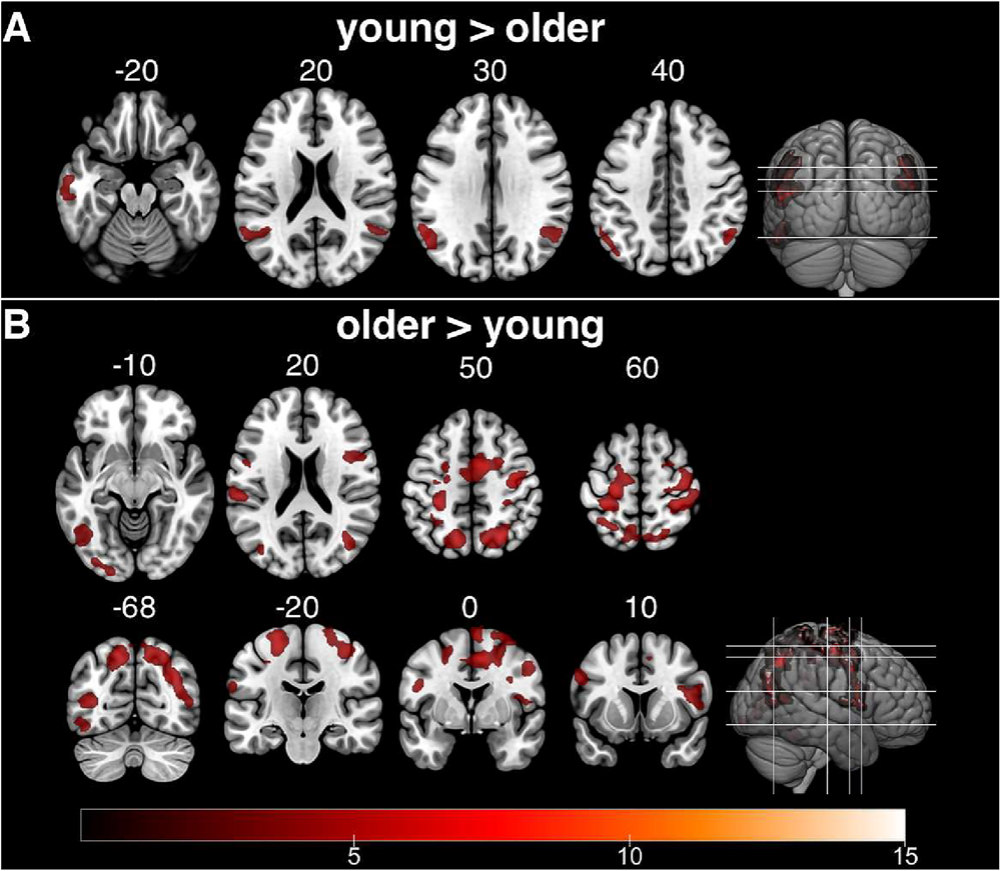
**Main effects of group (young vs. older participants) in the fMRI data**. (A) The significant clusters for the main effect of young > older participants are shown on axial slices and a surface rendering of a standard brain viewed from the occipital pole. (B) The significant clusters for the main effect of older > young participants are depicted on axial and coronal slices and a surface rendering viewed from the right. All clusters shown survived a statistical threshold of *p* < 0.05, family‐wise error (FWE) corrected for multiple comparisons at the cluster level using a cluster‐defining threshold of *p* < 0.001, uncorrected.

**TABLE 1:** Full factorial model: brain regions showing significant increases of the BOLD response for the main factors group (young and older participants) and condition (sequence and random blocks) and their interaction.

Brain region	Hemisphere	MNI coordinates				
		*x*	*y*	*z*	*t*‐score	Cluster size
**Young > older participants**						
Inferior parietal lobule	Left	−58	−56	30	5.16	806
	Right	54	−58	42	5.59	546
Middle temporal gyrus	Left	−62	−20	−20	4.10	296
**Older > young participants**						
Precentral gyrus	Right	28	−6	72	6.97	5079
		54	2	40	4.47	5079*
Inferior parietal lobule	Left	−60	−26	26	6.28	476
Superior parietal lobule	Left	−12	−68	50	5.45	2190*
	Right	28	−64	50	5.94	2190
Middle occipital gyrus	Left	−42	−70	10	5.16	685
Inferior frontal gyrus	Left	−52	8	32	4.95	263
	Right	46	8	16	4.73	497
Inferior temporal lobe	Left	−34	−50	−20	4.51	657
**Sequence > random blocks**						
Pre‐/Postcentral gyrus	Left	−34	−22	54	15.27	30,504
Intraparietal sulcus	Left	−28	−46	48	14.82	30,504*
	Right	28	−48	46	10.22	30,504*
Anterior precentral gyrus	Left	−26	−10	56	13.64	30,504*
	Right	28	−6	50	12.01	30,504*
Juxtapositional lobule cortex/Supplementary motor cortex	Left	−6	−4	56	13.03	30,504*
Occipital lobe	Left	−44	−74	2	12.18	30,504*
	Right	48	−66	0	11.44	30,504*
Cerebellum	Left	−32	−54	−26	8.32	30,504*
Vermis	Right	10	−56	−14	7.61	30,504*
Striatum	Left	−22	−2	6	7.73	1267
	Right	22	4	6	6.72	696
Superior temporal lobe	Right	50	−38	14	5.98	326
**Random > sequence blocks**						
Medial occipital lobe		8	−82	18	12.97	26,207
Posterior cingulate gyrus	Left	−8	−38	42	8.17	26,207*
	Right	6	−34	44	8.31	26,207*
Insula	Right	36	−14	18	8.13	26,207*
Posterior parietal cortex	Left	−44	−76	34	7.00	26,207*
	Right	52	−68	30	6.01	26,207*
Prefrontal cortex	Left	−52	32	14	6.48	1477
Inferior temporal lobe	Left	−52	−4	−16	6.22	26,207*
	Right	56	−2	−18	5.50	26,207*
Medial temporal lobe	Left	−26	−40	−10	6.11	26,207*
	Right	28	−34	−10	6.18	26,207*
Anterior cingulate cortex		0	36	16	5.67	2310
**Interaction term: (sequence > random blocks) for older participants > (sequence > random blocks) for young participants**						
Postcentral gyrus	Left	−30	−34	60	4.50	394
Precentral gyrus	Left	−22	−22	60	4.49	394*

*Note*: The coordinates in MNI space refer to the maximally activated voxel within an activation cluster. Cluster size indicates the extent of the activated cluster by the number of significant voxels (*k*_E_). Asterisks indicate submaxima within a given cluster. The listed clusters survived the statistical threshold of *p* < 0.05, family‐wise error (FWE) corrected for multiple comparisons at the cluster level based on a cluster‐defining threshold of *p* < 0.001, uncorrected.

#### 3.3.2 Main Effect of Condition

Comparison of sequence with random blocks (contrast sequence > random, Table 1 and Figure S1A) revealed activation of an extensive bilateral motor network, consisting of primary sensorimotor cortex (SM1, in the pre‐ and postcentral gyri), dorsal premotor cortex (in the anterior precentral gyrus), striatum (especially putamen), parieto‐occipital, and cerebellar areas, as well as the supplementary motor area (SMA, in the juxtapositional lobule cortex). Besides, the activation of the right superior temporal lobe was detected. The inverse contrast (random > sequence, Table 1 and Figure S1B) showed higher activity in the anterior and posterior cingulate cortex, the medial occipital lobe, the posterior parietal and temporal regions bilaterally, the right insula, and the left prefrontal cortex.

#### 3.3.3 Interaction Group × Condition

The interaction between the between‐subject factor group and the within‐subject factor condition was expected to reveal the brain areas associated with age effects on procedural learning. Although the interaction term of sequence > random blocks for older participants versus sequence > random blocks for young participants yielded a significant activation of the left sensorimotor cortex (Figure 4A, Table 1), there was no significant cluster associated with the inverse interaction term (i.e., sequence > random blocks for young participants vs. sequence > random blocks for older participants). To further explore this significant interaction, we extracted the beta values of the left sensorimotor cortex cluster revealed by the interaction term and performed post hoc *t*‐tests to compare sequence to random blocks. For both groups, beta values for sequence blocks were higher compared to random blocks (young: *t*(144.13) = −2.19, *p* < 0.05, older: *t*(134.32) = −5.92, *p* < 0.001).

**FIGURE 4:**
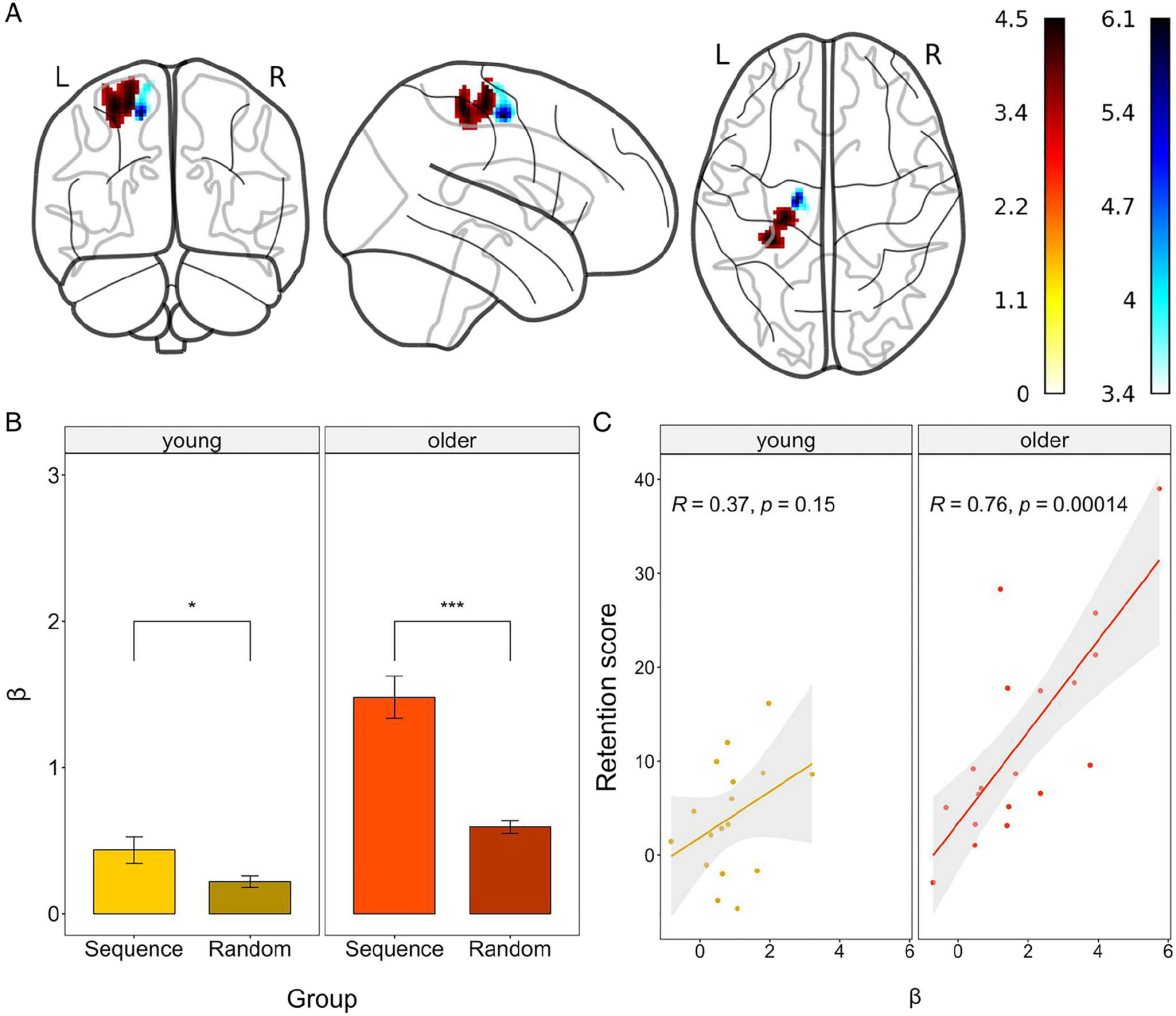
**Interaction of the factors group (older > young) and condition (sequence > random) in the fMRI data on Day 1 (A and B) and the results of the whole‐brain regression analysis of the retention score on Day 2 based on the fMRI contrast sequence > random on Day 1 (A and C)**. (A) The significant cluster associated with the group by condition interaction is depicted in a schematic brain in a yellow–red color scheme and was located in the left sensorimotor cortex. This cluster survived a statistical threshold of *p* < 0.05, family‐wise error (FWE) corrected for multiple comparisons at the cluster level using a cluster‐defining threshold of *p* < 0.001, uncorrected. The cluster with the significant peak voxel of the whole‐brain regression analysis is depicted in a blue color scheme and was located in the (dorsal) premotor cortex. This cluster survived a threshold of *p* < 0.05, family‐wise error (FWE) corrected at the voxel level. Color bars indicate *t*‐values. (B) Mean beta values of the significant cluster in the left sensorimotor cortex associated with the group by condition interaction showing that the significant interaction was due to a differential increase of beta values in sequence blocks (compared to random blocks) for the older adults (red colors), when compared to the corresponding beta‐value difference in the young adults (yellow colors; *p < 0.05, ***p < 0.001). (C) Correlation between the beta values of the peak voxel of the cluster located in the (dorsal) premotor cortex obtained from the aforementioned whole‐brain regression analysis with the retention score for young (yellow) and older (red) participants, separately.

Notably, the significant interaction was due to a differential increase of the beta values in sequence blocks (compared to random blocks) for the older participants, compared to the corresponding beta‐value difference in the young participants (see Figure 4B). These results correspond well to the interaction mentioned earlier between the effects of group and condition on the RTs at the behavioral level (see Section 3.2). The corresponding linear contrasts illustrating the interaction are shown in Figure 5.

**FIGURE 5:**
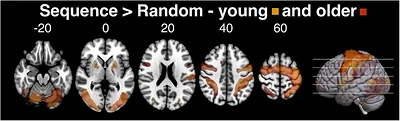
**Main effects of condition (sequence > random blocks) for both groups in the fMRI data**. The significant clusters for the main effect of sequence > random blocks are shown for young (orange) and older (red) participants on axial slices and a surface rendering of a standard brain viewed from the left side. All clusters shown survived a statistical threshold of *p* < 0.05, family‐wise error (FWE) corrected for multiple comparisons at the cluster level using a cluster‐defining threshold of *p* < 0.001, uncorrected.

#### 3.3.4 Regression Analysis

The whole‐brain regression analysis for the contrast sequence > random revealed a significant association between the behavioral retention score as assessed on Day 2 and the activity of the left (dorsal) premotor cortex during procedural learning on Day 1 (Table 2). In addition, the individual beta values of this premotor activation correlated significantly with the corresponding retention score for older (*R* = 0.76, *p* < 0.001) but not for young participants (*R* = 0.37, *p* = 0.15; both Figure 4C). Fisher's *z*‐test revealed that the correlation was stronger among the group of young participants than among the group of older participants (*z* = 1.7, *p* < 0.05).

**TABLE 2:** Regression analysis: brain regions showing a significant correlation of the BOLD response for the contrast sequence > random blocks (measured on Day 1) with the behavioral retention score on Day 2.

Brain region	Hemisphere	MNI coordinates				
		*x*	*y*	*z*	*t*‐score	Cluster size

Precentral gyrus	Left	−16	−14	54	6.05	139
		−12	−16	70	4.04	139*

*Note*: The coordinates in MNI space refer to the maximally activated voxels within an activation cluster. Cluster size indicates the extent of the activated cluster by the number of significant voxels (*k*_E_). Asterisks indicate submaxima within a given cluster. The listed cluster survived the statistical threshold of *p* < 0.05, family‐wise error (FWE) corrected for multiple comparisons at the voxel level.

## 4 Discussion

Our objective was to assess age‐related effects on procedural learning and retention, and whether core motor areas are involved in these processes. At the behavioral level, older adults showed a greater RT difference between sequence and random blocks on Day 1. Further, older adults showed even better retention of the learned motor sequence than younger adults. These findings align with previous studies showing similar procedural learning capabilities across age (Bhakuni and Mutha 2015; Daselaar et al. 2003; Frensch and Miner 1994; Gaillard et al. 2009; Howard and Howard 1989, 1992; Meissner et al. 2016). A floor effect among young participants could explain the larger nominal RT difference observed among older participants on Day 1. Although older participants decreased their mean RTs across sequence blocks, young participants’ mean RTs were already swift in the first sequence blocks, leaving little room for further improvement.

Consolidation of motor (sequence) knowledge is supposed to deteriorate in aging, as many studies have shown, also using the SRT task (Brown et al. 2009; Nemeth et al. 2010; Nemeth and Janacsek 2011; Roig et al. 2014; Spencer et al. 2007; Wilson et al. 2012). Surprisingly, the retention of motor sequence knowledge assessed with the current modified SRT task was even better in older than in younger adults. As the current modified SRT task comprised—in contrast to SRT task versions used in previous studies—direct (i.e., on each trial) feedback to our participants, our data may suggest that the observed better retention in older participants was driven by the direct feedback given during the initial procedural learning, from which the older participants seem to have benefitted more than the young participants. As the older subjects of the current study performed worse in some neuropsychological tests, better performance in the modified SRT task cannot be explained by having selected older participants with extraordinary memory.

Combining neural and behavioral data in a whole‐brain regression analysis, including both young and older participants, a significant correlation between left premotor cortex activity (i.e., in the hemisphere contralateral to the right hand performing the modified SRT task) and retention after procedural learning was revealed. Note that behavioral data from the day after the actual fMRI scanning session entered the regression analysis. Interestingly, beta values extracted from the peak voxel of this whole‐brain regression analysis correlated with the retention score only in older participants. This extends findings of studies in young participants showing that task‐dependent activity during procedural learning can predict subsequent consolidation (Albouy et al. 2008, 2013, 2015; Steele and Penhune 2010) by providing evidence for a specific activation of the premotor cortex in older subjects. The most parsimonious explanation for this association is that the feedback applied in the current modified SRT task may have led to stronger recruitment of the contralateral left premotor cortex, resulting in enhanced retention. This interpretation aligns with studies showing the importance of premotor cortical regions for different stages of procedural learning during the SRT task (Hardwick et al. 2013) and, more specifically, with studies demonstrating enhanced functional connectivity of the premotor cortex after procedural learning, particularly when feedback was applied during the learning phase (Steel et al. 2019).

Furthermore, older participants showed more robust recruitment of the left sensorimotor cortex (SM1) during procedural learning. These results go beyond previous reports, showing activation of a similar network of brain regions in young and older adults during an SRT task (Daselaar et al. 2003). According to the compensation hypothesis, over‐recruitment, specifically of SM1 (Heuninckx et al. 2008; Mattay et al. 2002), compensates for age‐related deficits in motor control due to structural, biochemical, or functional changes. Thus, the current data suggest that stronger activation of the left SM1 enabled our older participants to achieve procedural learning performance in the modified SRT task similar to that of young participants.

Our study is limited by a reduced sample size, as 11 participants had to be excluded due to issues during imaging, task execution, or clinical aspects. Still, the remaining sample was thoroughly controlled for potential confounders and covered a wide age range. At the behavioral level, a floor effect in RTs may have occurred in young adults. However, significant retention was observed in both groups, suggesting that the fast RTs in young adults did not impede subsequent retention. Hauptmann and colleagues 2005 argue that, in their study, consolidation occurred only “when practice continued to the point at which within‐session performance leveled off (saturation)” (Hauptmann and colleagues 2005). While not directly addressing retention, this finding suggests that the degree of stabilization achieved during training influences later offline effects. Furthermore, we assessed only early retention after a 24‐h interval.

Another limitation of our study is the lack of data on sleep quality, as sleep is known to affect motor memory consolidation and retention (Frazer et al. 2021; King et al. 2017; Leal et al. 2025) and aging changes sleep macro‐ and microstructure (Evans et al. 2021; Mander et al. 2017). However, given that the data indicated even greater retention in older adults, sleep‐related factors do not appear to be a major confound in our study. With respect to the imaging data, the analysis based on extracted beta values from the peak voxel of the whole‐brain regression represents a post hoc exploration intended solely to disentangle the contribution of each age group to this effect. Given these limitations, confirmatory studies with larger sample sizes that directly examine the relationship between dorsal premotor cortex activity and retention as a function of age, as well as studies assessing long‐term retention, are warranted to confirm and extend our findings.

## 5 Conclusion

In a modified SRT task with direct feedback, older participants showed stronger activation of the contralateral sensorimotor cortex during procedural learning. Moreover, older subjects showed a better retention of motor sequence knowledge (associated with an increased premotor activity), potentially due to the application of feedback during procedural learning in the current SRT task.

Besides the direct application of these findings for improving motor performance in healthy older adults, the current results may also be relevant for the development of novel strategies to enhance motor performance in patients with acquired motor deficits, for example, after a stroke.

## Author Contributions

**Christopher E. J. Doppler**: conceptualization, methodology, software, data curation, investigation, validation, formal analysis, visualization, project administration, writing – original draft. **Linda Meyer**: conceptualization, methodology, data curation, investigation, validation, formal analysis, project administration, writing – review and editing. **Ralph Weidner**: conceptualization, methodology, resources, writing – review and editing. **Peter H. Weiss**: conceptualization, methodology, validation, formal analysis, supervision, resources, writing – review and editing. **Gereon R. Fink**: conceptualization, methodology, supervision, funding acquisition, resources, writing – review and editing.

## Funding

Christopher E. J. Doppler is supported by the Clinician Scientist Program (CCSP)/Faculty of Medicine/University of Cologne, funded by the Deutsche Forschungsgemeinschaft (DFG, German Research Foundation, FI 773/15‐1). Gereon R. Fink and Peter H. Weiss are funded by the Deutsche Forschungsgemeinschaft (DFG, German Research Foundation)—Project‐ID 431549029—SFB 1451. Open access publication funded by the Deutsche Forschungsgemeinschaft (DFG, German Research Foundation)—Project‐ID 491111487.

## Ethics Statement

The ethics committee of the Faculty of Medicine, University of Cologne, had approved the study.

## Consent

We obtained written informed consent from all participants in accordance with the Declaration of Helsinki.

## Conflicts of Interest

The authors declare no conflicts of interest.

## Supporting information




**Supplementary Figure**: brb371845‐sup‐0001‐FigureS1.docx

## Data Availability

The data that support the findings of this study are available from the corresponding author upon reasonable request.
