# Supplementary material for: Procedural Learning and Retention in Older Subjects: The Contribution of Sensorimotor and Premotor Cortex
Source: Brain Behav. 2026 Oct 16;16(10):e71845. doi: 10.1002/brb3.71845 (PMC13674733; doi:10.1002/brb3.71845)
Supplement: Supplementary file 1 [file BRB3-16-e71845-s001.docx]

**Supplementary Figure S1: *Main effect of condition (sequential vs. (pseudo-)random order of cues) in the fMRI data***

Significant clusters for the contrast sequence > random blocks (A) and the inverse contrast (random > sequence, B) are shown on axial and coronal slices of a standard brain. All clusters shown survived a statistical threshold of p < .05, family-wise error (FWE) corrected for multiple comparisons at the cluster level using a cluster-defining threshold of p < .001, uncorrected.
